# Low palaeoelevation of the northern Lhasa terrane during late Eocene: Fossil foraminifera and stable isotope evidence from the Gerze Basin

**DOI:** 10.1038/srep27508

**Published:** 2016-06-08

**Authors:** Yi Wei, Kexin Zhang, Carmala N. Garzione, Yadong Xu, Bowen Song, Junliang Ji

**Affiliations:** 1 Faculty of Earth Sciences, China University of Geosciences (Wuhan), Wuhan, Hubei, China; 2 State Key Laboratory of Biogeology and Environmental Geology, China University of Geosciences, Wuhan 430074, Hubei, China; 3 Department of Earth and Environmental Sciences, University of Rochester, Rochester, NY, USA; 4 Institute of Geological Survey, China University of Geosciences, Wuhan 430074, Hubei, China

## Abstract

The Lhasa terrane is a key region for understanding the paleoelevation of the southern Tibetan Plateau after India-Asia collision. The Gerze Basin, located in the northern part of the Lhasa terrane, is a shortening-related basin. We discovered *Lagena laevis* (Bandy) fossils in upper Eocene strata of the Gerze Basin. This type of foraminifera is associated with lagoon and estuarine environments, indicating that the northern part of the Lhasa terrane was near sea level during the late Eocene. We speculate that these foraminifera were transported inland by storm surges to low elevation freshwater lakes during times of marine transgressions. This inference is consistent with the relatively positive δ^18^O values in carbonate from the same deposits that indicate low palaeoelevations close to sea level. Considering the palaeoelevation results from the nearby Oligocene basins at a similar latitude and the volcanic history of the Lhasa terrane, we infer that large-magnitude surface uplift of the northern Lhasa terrane occurred between late Eocene and late Oligocene time.

The Tibetan Plateau is widely considered to be the result of shortening related to convergence both preceding and post-dating the initial collision between the Indian and Eurasia plates at 65–50 Ma^1^,^2^,^3^. However, with the advent of quantitative palaeoelevation methods, the association between the timing of crustal thickening and the timing of surface uplift of mountain belts has been challenged^4^,^5^. Consensus has not been achieved regarding the surface uplift history of the Tibetan Plateau. Stable isotope paleoelevation estimates from the Nima and Lunpola basins indicate paleoelevations similar to modern elevations by 26 Ma^5^,^6^ along the Bangong suture (Fig. 1). Wang *et al.*^7^ proposed that surface uplift of the southern and central Tibetan Plateau (Lhasa and southern Qiangtang terranes) occurred by 40 Ma, based primarily on stratigraphy and thermochronology results. Arguing for an Eocene “proto-Tibetan Plateau”, Kapp *et al.*^8^ suggested that the collision between the Lhasa terrane and the Qiangtang terrane resulted in crustal shortening of the Lhasa terrane that may have raised southern Tibet to an elevation of 3–4 km during the Cretaceous, prior to India-Asia collision. This inference of a high proto-Tibetan plateau prior to India-Asia collision is reinforced by paleoelevation reconstructions of the Linzhou Basin, which suggest that the Gandese magmatic arc region in the southern Lhasa terrane attained an elevation of ~4500 m by the time of India-Asia collision^9^. In contrast, Hetzel *et al.*^10^ performed thermal modelling of (U-Th)/He ages of apatite and zircon, as well as apatite fission track data, from the northern Lhasa terrane, and inferred that peneplanation of the northern Lhasa terrane occurred at least 3–4 km lower than its current elevation of ~5300 m. Moreover, the formation of the peneplain at low elevation was completed by ca. 50 Ma, and the resistant bedrock surface has undergone slow erosion since then. Zhang *et al.*^3^ also argued that the presence of the proto-Tibetan Plateau does not match the sedimentary characteristics and overall topography during the suggested time period based on a detailed summary of 121 geological maps (1:250000) of the Tibetan Plateau area, and they estimated that the average palaeoelevation in central Tibet was ~500 m at ca. 34 Ma.

Based on conflicting evidence for high paleoelevations from sparse stable isotope studies and low paleoevelations from fossil, stratigraphic, and thermochronology/thermal modelling studies, it remains unclear whether the Lhasa terrane was at a high elevation at and following the collision of the Indian and Eurasian plates. To determine the paleoelevation history of the Lhasa terrane, we collected both fossil and stable isotope data from a late Eocene section in the Gerze Basin. We provide age information for these deposits, based on magnetostratigraphy with additional age constraints from fossils and zircon U-Pb dates.

## Results

### Lithostratigraphy

Eocene strata in the northern Lhasa terrane are defined as the Dingqinghu Formation. This study is focused on the upper part of the Dingqinghu Formation, located at 31°54.655′N, 85°7.693′E (4618 m), with a thickness of 330 m (Fig. 2a). The base of the section is marked by a thick layer of conglomerate, and the top of the section is the core of a syncline. The lower part (layers 1–26) of the section consists of medium- to thickly bedded brown conglomerate, pebbly sandstone, and cross-stratified sandstone. Occasionally interbedded with clastic deposits are thick beds of grey bioclastic limestone that contain freshwater ostracods, gastropods and charophyte fossils. These facies suggest that the depositional environment for the lower part of the section was lacustrine to marginal lacustrine. The upper part (layers 27–86) mainly consists of thin-medium beds of grey limestone intercalated with grey shale and two pebbly sandstone layers (<20 cm thick). These facies suggest that the depositional environment of the upper part of the section was a stable shallow lacustrine environment. The bedding thins upward in the section, suggesting a transition to deeper water environments.

### Chronostratigraphy

We use three different methods to constrain the ages of the investigated section: detrital zircon U-Pb ages, index fossils and magnetostratigraphy. We collected three detrital zircon samples from layers 1, 12, and 25, which in total yielded 198 analysis points for the selected zircon grains. The three youngest detrital zircon grains yielded a mean age of ca. 42 Ma (Fig. 2b), and the youngest zircon population provides the maximum depositional age for our section.

Previous research has shown that the ostracoda genus *Austrocypris* is an important index fossil that was short-lived in the late Eocene^11^,^12^. This genus is widely observed in the lower member of the late Eocene Xiaganchaigou Formation in the Qaidam Basin^11^,^12^,^13^ and in the fourth member of the Shahejie Formation in the Dongpu depression^14^ and Bohai Sea Basin in eastern China^15^. Their extinction may correspond with the Eocene-Oligocene climate transition that occurred at ca. 34 Ma^12^,^16^. This ostracoda is observed in our study section between layer 8 and layer 76 and can be used for regional stratigraphic correlation to bracket the timing of events. Moreover, *Gyrogona qianjiangica,* which is another species of chara, is an index fossil of the late Eocene Xiaganchaigou Formation^13^ that occurs from layer 9 to layer 83.

Based on the above late Eocene fossil age constraints, the age of this section is bracketed between 42 Ma and 34 Ma. A total of 197 oriented palaeomagnetic samples were collected in our section to acquire high-resolution (average ~1.6 m) magnetostratigraphic data (see Supplementary Table S1 and Fig. S1). The results can be correlated with the standard polarity time scale 2004 (GTS 2004)^17^, which covers an age range of 39.4–35.7 Ma.

### Foraminifera fossils

We found a foraminifera fossil shell in layer 2 and five foraminifera fossil shells in layer 23 (Fig. 2c). The lithology of layer 2 is purple-red medium-thick bedded laminated siltstone, whereas the lithology of layer 23 is purple-red thinly bedded massive mudstone.

These fossils are well preserved with obvious identifying characteristics, indicating that they are the same species. The fossils are globular, ovoid, and widest just below the middle section and presented circular cross sections. Additionally, they have smooth rounded bases, short, round, tapering necks approximately one-ninth the length of the body, and smooth, finely perforated walls. The plesiotype length is 0.23 mm, and the diameter is 0.20 mm. The elemental composition was detected via an energy dispersion spectrometry analysis, and the results indicate that they are mainly calcareous (Fig. 2c). Based on the neck shape, clear-walled burrow structure and standard calcareous composition, these fossils are identified as *Lagena laevis* Bandy. This species belongs to the *Lagena* genus, Nodosariacea family, and Rotaliina order and has been found in the Eocene Oujiang Formation in the East China Sea shelf and late Eocene strata in Alabama, USA^18^. *Lagena laevis* Bandy is a benthic^19^ warm water foraminifera species that occupies a narrow range of salinities and can adapt to a strong hydrodynamic environment. This organism occurs in estuaries or coastal bay sedimentary environments and has been reported in offshore gulfs around the world^20^,^21^,^22^,^23^.

### Oxygen isotope paleoelevation reconstructions

126 limestone samples were collected for oxygen isotope analysis to reconstruct palaeoelevation. All of the carbonate results are reported in δ^18^O_c_ (VPDB-Vienna PeeDee belemnite). Evaporation during deposition and post-depositional diagenesis are two major factors that can influence oxygen isotope values, resulting in biased paleoelevation reconstructions, and thus we carry out a detailed evaluation of these factors. Analysis of diagenetic phases provides insights into whether the δ^18^O_c_ values of the primary micritic carbonates have been modified^24^. Thin section analyses show that the lacustrine carbonates from layers 1–30 of our section contain significant (>50%) coarse-grained spar that is characterized by extensive dissolution cavities filled with sparry calcite (Fig. 3a,b). However, the micrites from layers 31–86 of our section generally preserve primary pore spaces (Fig. 3c) that are usually filled with fine-grained blocky calcite textures associated with early diaganesis, when pore fluids reflect surface water compositions and temperatures. Some samples show vein calcite (Fig. 3d). To determine the possible influence of veins and coarse-grained blocky calcite that filled dissolution cavities, primary micritic carbonate and late stage diagenetic phases were sampled using a microdrill to separate the micrite from the sparite for analysis. Sparry calcite within veins and dissolution cavities show δ^18^O_c_ values of −20.8‰ to −5.3‰ (see Supplementary Table S2), whereas micrite δ^18^O_c_ (VPDB) values are much more positive, ranging from −8.1‰ to −2.1‰, with an average of −5.1 ± 1.1‰ (1σ) (see Supplementary Table S3).

In addition to diagenesis, evaporation is another factor that can influence the δ^18^O values of lake water and carbonate. In a hydrologically open system, oxygen isotopes are less likely to be affected by evaporation. δ^13^C and δ^18^O values that present variation coefficients of r >0.7 are considered to indicate a closed lake system or long-term residence times^25^. Lacustrine micrite from layers 31–86 show poor correlations between δ^18^O_c_ and δ ^13^C_c_ values (r = 0.0173) (Fig. 3e), which implies a hydrologically open condition. Sparite from layers 1–30 shows a distinct isotopic composition from micrite, indicating that micrite is a faithful recorder of the primary isotopic composition of carbonates. The very negative δ^18^O_c_ end-member of about −20‰ for sparite indicates that these diagenetic carbonates either precipitated from meteoric water with a low δ^18^O value, consistent with modern high altitude precipitation, or from waters of similar composition to what precipitated the micrites, but at burial temperatures higher than surface temperature^24^.

## Discussion

### Paleogeography inferences from foraminifera

Although foraminifera fossils mainly occur in marine strata, they are also distributed in Late Quaternary strata located hundreds of kilometres away from current shorelines associated with marine transgressions^26^,^27^. Inland basins that have little contact with the ocean may also contain a small amount of foraminifera^28^,^29^ associated either spatially or temporally with marine sources. The airborne colonization of these inland water bodies via migratory birds has been suggested by previous research, although this type of colonization is occasional and does not result in selectivity of species. The foraminifera fossils from our study section all belong to the same species and have a relatively high abundance. This species has a nearly hollow spherical shape, and its selectivity is likely related to the large specific surface area of its structure, which facilitates its transport during storm surges. Research has also shown that foraminifera from older marine sources can adapt to and thrive in suitable inland saltwater environments^30^. However, in the investigated section, the underlying and overlying lacustrine limestone strata contain rich chara and lacustrine ostracoda and represent clear and shallow lacustrine environments. Additionally, this species of foraminifera has not been previously reported in older marine strata across the Lhasa terrane and thus is unlikely to have survived from older marine sources.

**Figure 1:**
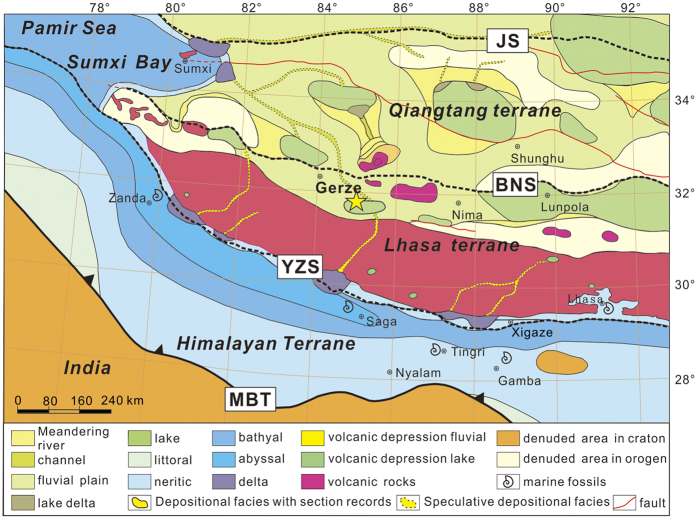
Eocene palaeogeography of Tibet and marine fossil locations (modified from Zhang *et al*.)^32^,^37^,^60^. Major suture zones or thrust faults in the plateau include main boundary thrust (MBT), Yarlung Zangbo suture (YZS), Bangong–Nujiang suture (BNS), and Jingshajiang suture (JS).

The *Lagena laevis* Bandy foraminifera could have been transported hundreds of kilometres inland from the ocean by marine transgression or by large storms if a passageway was available^31^. Two possible passageways include the fluvial volcanic depression through Gangdese volcanic arc, which is connected to the Himalayan Sea in southern Tibet, and the channels that occur through the coastal-alluvial plain connected to Sumxi Bay of the Pamir Sea in western Tibet (Fig. 1)^32^. Both the Himalayan Sea and Pamir Sea belong to the Neo-Tethys Sea.

1. Passageway from the Himalayan Sea. The Palaeogene Himalayan seaway in Tibet was an east-west trending remnant sea that covered southern Tibet and the Gangdese areas. Burrard^33^ and Douville *et al.*^34^ discovered Palaeogene Himalayan marine strata in Gamba and Tingri (Fig. 1), and researchers have also discovered many foraminifera, ostracoda and chara fossils in these areas and conducted further studies of the marine strata^35^,^36^. Previous research has shown that the Zongpubei Formation in Gamba and Tingri contain the youngest documented marine strata^37^,^38^. The Zongpubei Formation is dated to the early Priabonian stage of the Eocene (ca. 35 Ma) and in both areas yields larger foraminifera, including *Nummulites willcoxi* and *Nummulites manila*, and other smaller foraminifera. The strata at the top of the Zongpubei Formation in these areas are cut by faults and are sparsely outcropping. However, the deposition in southern Tibet was stable during the Eocene^37^,^38^, thus, the latest time of regression may have been after the early Priabonian stage of the late Eocene. Furthermore, float rocks that contain rich foraminifera have been found in both Niumagou and Zhongba, which are located to the north of Lhasa City. The foraminifera are identified as *Keramosphaera tergestina* (Stache), which only lived during the Palaeocene-Eocene^39^. Although the fossil positions are not clear, seawater certainly reached the area near Lhasa City during the early Tertiary. Deltaic deposits have been documented in several Eocene stratigraphic sections along the southern margin of the Gangdese volcanic arc^32^, indicating a fluvial drainage basin sourced in the Gandese volcanic arc. This palaeogeography could have enabled the foraminifera to be transported through channels to be deposited in the Gerze Basin during transgressions and/or extreme storms if the Gerze Basin and the fluvial system was located at a low elevation. However, previous research suggested that portions of the magmatic arc were at high elevation at this time^9^, which seems inconsistent with a low elevation fluvial volcanic depression through the Gangdese volcanic arc.
2. Passageway from Sumxi Bay. Gulf lagoons developed in the Sumxi area at the easternmost part of the Pamir Sea during the Eocene preserved as the Kashi Group^40^. The Kashi Group records three transgression-regression cycles in the Tarim Basin^41^,^42^ that occurred northwest of the Sumxi area. Among these transgressions, the last marine incursion occurred at 41–38 Ma (late Eocene), overlapping the time frame during which foraminifera appeared in the Gerze Basin. Sedimentary facies in the Sumxi area indicate a neritic environment, with several delta systems located along the eastern margin of Sumxi bay. An extensive area in the southwestern part of Sumxi Bay contained an alluvial plain and lakes^32^,^43^ (Fig. 1). We infer that rivers connected the Sumxi Bay to inland lakes, similar to the Yangtze River in eastern China and the Mississippi River in the USA. These channels may have enabled eastward intrusions of seawater during large transgression events, which could have transported the foraminifera *Lagena laevis* Bandy from an estuary environment to the Gerze Basin on the northern margin of the Lhasa terrane. This process requires that this region was located at low paleoelevation to allow for occasional intrusion if marine water containing foraminifera.

### Evaluation of palaeoelevation

Based on the discovery of foraminifera fossils, the Gerze Basin appears to have been close to sea level during the late Eocene (ca. 39 Ma). Oxygen isotopes from lacustrine micrites enable evaluation of the paleoelevation of this region. Comparisons between the δ^18^O values of modern surface water and palaeo-surface waters can be used to evaluate temporal changes in the surface elevation and climate^44^. Study of modern surface water and air mass trajectories across the Tibetan Plateau shows that most of the moisture that reaches the southern plateau (Lhasa terrane and the area to the south) is derived from a southern source region^45^. The δ^18^O values of modern surface water in the southern plateau, including the Lhasa terrane, conform well with a simple Rayleigh distillation model of vapor mass depletion in ^18^O, indicating that stable isotopes of meteoric water provide reliable reconstructions of palaeoelevation^44^.

We assume that 25 ± 10 °C reflects a reasonable temperature uncertainty for lacustrine carbonate precipitation that could account for the overall warmer Eocene conditions and low latitude^46^, even at moderate to high paleoelevations. We then used the mean δ^18^O_c_ (VPDB) of lacustrine micrites of −5.1 ± 1.1‰ (1σ) and the temperature-dependent fraction equation between water and calcite^47^ to calculate the δ^18^O_psw_ (psw - paleo-surface water) relative to SMOW - Standard Mean Ocean Water of −2.8 ± 1.7‰. The 1σ uncertainty in this δ^18^O_psw_ estimate is determine by a bootstrap Monte Carlo approach that propagates errors associated with uncertainty in the temperature of carbonate precipitation, error in the temperature-fractionation equation^47^, and scatter in the δ^18^O values of lacustrine micrite. Because of global cooling and the build-up of the continental ice sheets, the oxygen isotope value of the sea water has changed by approximately +1‰ between the Eocene and the present day^48^. For comparison to modern meteoric water, the δ^18^O_psw_ value is therefore corrected to −1.8 ± 1.7‰. We compared this value of δ^18^O_psw_ to modern meteoric water isotope results (annual average weighted by precipitation amount) of the GNIP (Global Network of Isotopes in Precipitation) sites in Asia^49^. Considering the mean δ^18^O_psw_ of −1.8 ± 1.7‰, with values as low as −3.5‰ at 1σ, these values are observed in modern rainfall that occurred at 50 ± 600 m asl (Fig. 4). The high δ^18^O_psw_ values in Eocene deposits of the northern Lhasa terrane are therefore consistent with fossil foraminifera results, which both indicate low paleoelevations, close to sea level. This low palaeoelevation inference agrees with sedimentary characteristics and the overall pattern of deposition and erosion of the Tibetan Plateau during the Eocene^3^ (Fig. 5a).

The Nima Basin and Lunpola Basin are also located to the south of the Bangong-Nujiang orogenic belt at approximately the same latitude as the Gerze Basin. According to previous palaeoelevation study results, in the late Oligocene (~26 Ma), the Nima Basin had reached approximately 4,000 m^5^ and the Lunpola basin had reached 3,190 ± 100 m^50^,^51^. Combining these results with the results of our study suggests that the northern Lhasa terrane experienced surface uplift of at least 3,000 m over approximately 10 million years from the late Eocene to the late Oligocene.

Evidence from low temperature thermochronometers and the history of magmatism in the Lhasa terrane are consistent with a low paleoelevation of the northern Lhasa terrane in the late Eocene. Thermal modelling based on the (U-Th)/He ages of apatite and zircon, as well as apatite fission track data from the northern Lhasa terrane indicate that a peneplain formed at least 3–4 km beneath its current elevation of ~5300 m^10^. The formation of this low elevation peneplain was completed by ca. 50 Ma, and the resistant bedrock surface has undergone very slow erosion since then. Zircon age records^52^,^53^,^54^ indicate an approximately 10 million-year period of magmatic quiescence in the Lhasa terrane between ca. 40–30 Ma with a significant magmatic event occurring after ca. 30 Ma (Fig. 5b(1)). Widespread magmatism initiated with adakitic rocks by ~26 Ma, followed by potassic-ultrapotassic rocks by ~24 Ma^55^. The chemistry of these magmas has been attributed to break-off the Indian continental lithosphere that had subducted beneath the Lhasa terrane and/or convective removal of the Tibetan lithospheric mantle^55^,^56^. Ninety-six apatite fission track ages collected from the Lhasa terrane indicate three cooling age peaks at 22–15, 10–7, and 5–0 Ma, whereas the Palaeogene ages are more scattered^57^ (Fig. 5b(2)). Magmatic zircon ages and apatite fission track ages combined suggest minimal exhumation of the Lhasa terrane before the Oligocene. Additionally, widespread unconformities and related basal-conglomerates are dated at ca. 34 Ma, and alluvial fan and fluvial conglomerates resulting from rapid erosion are documented in the peripheral areas of the plateau, such as the Shimagou, Huoshaogou and Tala Formations along the north of the plateau^3^.

Fossil foraminifera and oxygen isotope studies of Eocene Gerze basin lacustrine deposits indicate that the northern Lhasa terrane resided at paleoelevations close to sea level between ~39 to 36 Ma. In combination with results from the Nima basin and Lunpola basin that indicate high paleolevations of ~4000 m by 26 Ma, we infer >3 km surface uplift of the northern Lhasa terrane between 36 Ma and 26 Ma. The temporal association between this large magnitude surface uplift and widespread adakitic and potassic-ultrapostassic magmatism across the Lhasa terrane after ~30 Ma suggests that this surface uplift event was associated with break-off Indian continental lithosphere and/or convective removal of the lithospheric mantle beneath the Lhasa terrane.

## Methods

### Detrital zircon age

**Figure 2:**
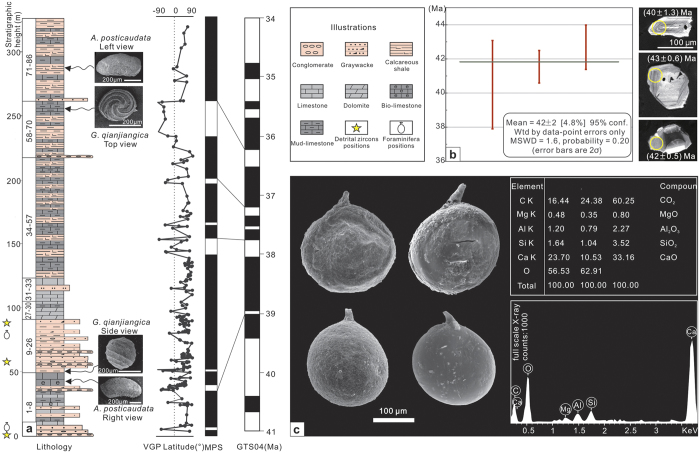
Generalized Gerze section in terms of lithology, fossils, and age. (**a**) Lithologic column, index fossils, and magnetostratigraphic results with VGP latitude against the stratigraphic level, and correlations with the geological time scale of GTS2004^17^. (**b**) Young detrital zircon ages of our study section, with CL photos at same scale (MSWD = mean standard weighted deviation). (**c**) Photos and energy dispersion spectrometry analysis of the foraminifera fossils (the top-left photo is from layer 2, and the others are from layer 23).

**Figure 3:**
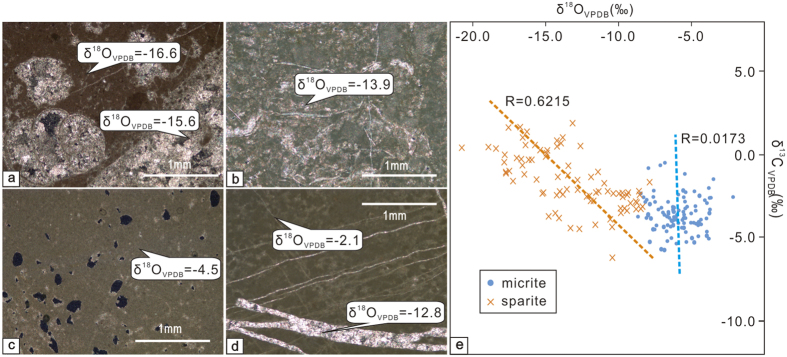
Photomicrographs and stable isotope results from Gerze section. (**a–d**) Photomicrographs from the Gerze section carbonates in the Lhasa terrane. All of the images were taken under cross-polarized light (VPDB-Vienna PeeDee belemnite). (**e**) Correlations between δ^13^C and δ^18^O from primary (micrite) and secondary diagenetic (sparite) carbonates.

**Figure 4:**
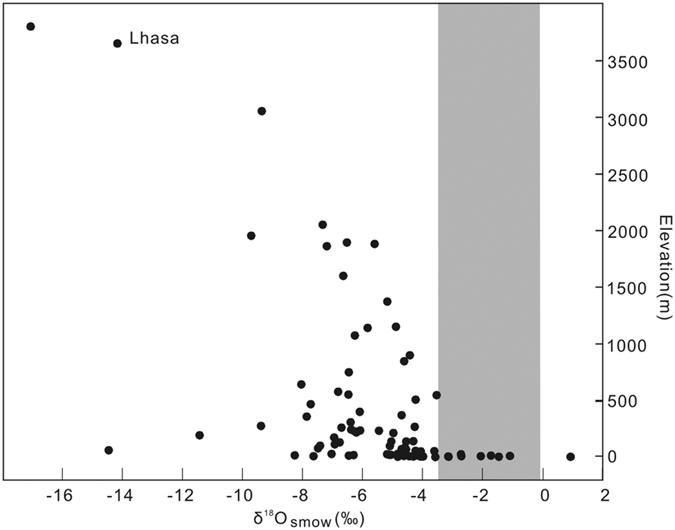
Weighted mean annual values of δ^18^O (SMOW - Standard Mean Ocean Water) from the Global Network of Isotopes in Precipitation sites of Asian regions (values from IAEA/WMO, 2012)^49^.

**Figure 5:**
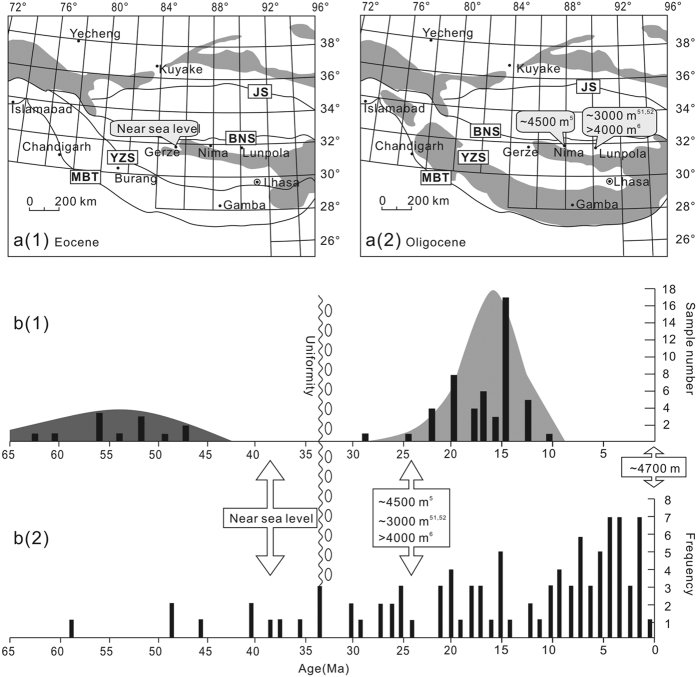
Paleogeographic and provenance response to surface uplift and exhumation of the Lhasa terrane. (**a**) Evolution of depositional areas (white) and denuded areas (grey) of the Qinghai-Tibetan Plateau and adjacent areas from (**a**(**1**)) the Eocene to (**a**(**2**)) the Oligocene modified from Zhang *et al.*^3^, (**b**(**1**)). Statistical distribution of zircon SHRIMP U-Pb ages for the Miocene adakitic rocks from southern Tibet modified from Mo *et al.*^52^, (**b**(**2**)). Histogram of apatite fission track ages in the Lhasa terrane^57^.

A total of 198 analysis points of selected zircon grains were analysed using a Neptune MC-ICP-MS (multicollector inductively coupled plasma mass spectrometer) (Thermo Fisher, Lafayette, Colorado, USA) with a UP193-FX ArF-excimer laser-ablation system (ESI, Sunnyvale, California, USA) at the Isotope Laboratory of Tianjin Center, China Geological Survey (CGS). Data were collected with a 35 μm spot and the ablation depth was 20–40 μm. Zircon 91500 was the external standard used for age calculation, NIST SRM 610 silica glass was the external standard used for calculation of concentrations, and Si was the internal standard. For ages younger than 1,000 Myr ago, the discordance is defined as (^207^Pb/^235^U–^206^Pb/^238^U)/ ^207^Pb/^235^U*100 and ^206^Pb/^238^U ages were adopted for them. All the ages of the three zircon grains in Fig. 2b present a low discordance degree (<10%).

### Magnetostratigraphy

A total of 197 oriented palaeomagnetic samples were collected in the field to acquire high-resolution magnetostratigraphy data. Because most of the palaeomagnetic samples originated from limestone, which contains small amounts of magnetic minerals, we used different methods to conduct the magnetism tests. Samples with NRM (natural remanent magnetization) ≥10^−5^ T were subjected to stepwise (averaging 17 steps) thermal demagnetization using a Thermal Demagnetizer (TD-48) (ASC Scientific Ltd, Carlsbad, California, USA). The heating routine is as follows: 150 °C/ 200 °C/ 250 °C/ 300 °C/ 350 °C/ 400 °C/ 450 °C/ 500 °C/ 525 °C/ 550 °C/ 585 °C/ 610 °C/ 625 °C/ 640 °C/ 660 °C/ 680 °C. Samples with NRM <10^−5^ T were subjected to stepwise (averaging 17 steps) alternating demagnetization using a D2000 & ARM Alternating Demagnetizer (2G Enterprises, Mountain View, California, USA). The demagnetization routine is as follows: heating up to 120 °C, then follow with alternating demagnetization steps as 30 mT/ 35 mT/ 40 mT/ 45 mT/ 50 mT/ 70 mT/ 100 mT/ 150 mT/ 200 mT/ 250 mT/ 300 mT/ 400 mT/ 500 mT/ 600 mT/ 700 mT/ 800 mT. Magnetic remanence was measured using a 2G superconducting magnetometer and JR6A two-speed rotation magnetometer (2G Enterprises, Mountain View, California, USA) housed in field-free space. The tests were performed at the Centre of Analysis and Testing, Institute of Earth Environment, Chinese Academy of Sciences. The characteristic remanence directions were determined using principal component analysis, and directions were analyzed using Fisher statistics.

### Microfossils

We collected 183 bulk samples for a microfossil analysis from the study section. A 15% solution of H_2_O_2_ was used for the sample preparation of microfossils from mudstone and siltstone. The heating-acid-digestion method with pure acetic acid (>99.5%) was used for the sample preparation of microfossils from limestone. More than 1300 ostracoda fossils and 6 foraminifera fossils were handpicked and sorted. We selected well-preserved specimens for photography using a scanning electronic microscope. An energy dispersion spectrometry analysis of the foraminifera fossils was performed at the State Key Laboratory of Geological Processes and Mineral Resources of the China University of Geoscience.

### Oxygen isotope

We collected 126 limestones samples from the bottom to the top of the cross section, and they were generally evenly distributed. The δ^18^O data are reported with respect to the V-PDB standard. Analyses of the stable isotope were performed at the SIREAL (Stable Isotope Ratios in the environment, Analytical Laboratory) of the University of Rochester using a “Delta Plus XP” mass spectrometer (ThermoFinnigan, San Jose, California, USA). δ^18^O_psw_ can be calculated from the δ^18^O_c_, which was fractionated from paleo-waters. The fractionation factor (α) is defined as: α_(calcite-water)_ = (1000 + δ^18^O_calcite_)/ (1000 + δ^18^O_water_). The equation^47^ to calculate (α) is: 1000lnα_(calcite-water)_ = 18.03(10^3^T^-1^)−32.42 (T in Kelvin). The equation^58^ to calculate δ^18^O_(SMOW)_ from δ^18^O_(VPDB)_ is: δ^18^O_(SMOW)_ = 1.03091 δ^18^O_(VPDB)_ + 30.91.

We assume 25 ± 10 °C as a reasonable lacustrine carbonate precipitation temperature to account for overall warmer conditions, the low latitude position of southern Tibet in Eocene^59^.

## Additional Information

**How to cite this article**: Wei, Y. *et al.* Low palaeoelevation of the northern Lhasa terrane during late Eocene: Fossil foraminifera and stable isotope evidence from the Gerze Basin. *Sci. Rep.* **6**, 27508; doi: 10.1038/srep27508 (2016).

## Supplementary Material

Supplementary Information
