# Supplementary material for: Low palaeoelevation of the northern Lhasa terrane during late Eocene: Fossil foraminifera and stable isotope evidence from the Gerze Basin
Source: Sci Rep. 2016 Jun 8;6:27508. doi: 10.1038/srep27508 (PMC4897749; doi:10.1038/srep27508)
Supplement: Supplementary Information [file srep27508-s1.pdf]

Supplementary Information

Low palaeoelevation of the northern Lhasa terrane during late Eocene: Fossil foraminifera and stable isotope evidence from the Gerze Basin

Yi Wei[1, 2], Kexin Zhang[1, 2], Carmala N. Garzione[3], Yadong Xu[1, 2], Bowen Song[4], Junliang Ji[1, 2]

[1] Faculty of Earth Sciences, China University of Geosciences (Wuhan), Wuhan, Hubei, China;

[2] State Key Laboratory of Biogeology and Environmental Geology, China University of Geosciences, Wuhan 430074, Hubei, China;

[3] Department of Earth and Environmental Sciences, University of Rochester, Rochester, NY, USA;

[4] Institute of Geological Survey, China University of Geosciences, Wuhan 430074, Hubei, China

*corresponding: K.X.Z. (kx_zhang@cug.edu.cn)

C.N.G. (carmala.garzione@rochester.edu)

Contents

Part 1 Supplementary Table S1-3

Part 2 Supplementary Figure S1

Supplementary Table S1 Paleomagnetism Data

Sample	Ds	Is	MAD	VGP	Deep
S33-84-2	330.2	65.8	5.3	62.31211	320.0
S33-84-1	302.2	64.0	6.9	43.96107	315.0
S33-82-2	331.0	4.1	4.0	49.55051	306.4
S33-81-1	285.2	62.3	2.8	31.71029	303.4
S33-78-1	281.0	7.3	15.1	11.27032	288.8
S33-77-1	293.5	-61.4	1.8	-6.1808	287.2
S33-76-2	325.5	37.2	15.0	57.31352	281.5
S33-75-3	38.2	-68.5	8.1	-0.12726	278.0
S33-75-1	12.0	3.4	4.2	57.75174	276.8
S33-71-2	296.5	20.1	5.5	27.88734	262.2
S33-71-1	48.4	26.7	14.5	42.49385	262.1
S33-70-2	157.2	-72.0	12.5	-60.4066	261.2
S33-70-1	169.9	-53.1	14.6	-81.3316	261.0
S33-69-1	226.5	-57.9	14.8	-51.8467	257.4
S33-68-1	169.0	-79.0	12.7	-52.6076	253.6
S33-66-2	236.0	-68.4	21.7	-45.1534	247.9
S33-64-1	183.2	-62.1	12.8	-78.2656	238.0
S33-62-3	254.6	-87.2	4.8	-33.2214	234.7
S33-62-2	93.0	31.0	16.2	6.286065	234.3
S33-61-3	280.8	-3.7	11.9	8.15902	230.9
S33-60-4	294.2	-61.5	9.7	-5.85253	226.2
S33-60-2	20.5	46.7	12.4	71.82935	222.4
S33-59-1	0.7	48.0	14.9	87.08069	219.0
S33-56-5	277.2	73.2	10.5	30.48855	216.0
S33-56-4	338.7	17.7	9.3	59.81052	215.5
S33-56-3	42.8	12.4	5.7	42.60422	214.0
S33-56-2	285.1	-57.9	15.2	-8.99967	213.0
S33-55-1	359.5	-0.2	4.3	57.9965	208.9
S33-52-3	343.9	23.4	8.0	65.36454	203.0
S33-52-2	69.6	3.6	9.4	18.20372	202.1
S33-52-1	260.3	-81.8	5.8	-33.1877	200.3
S33-51-1	228.1	-32.7	7.7	-44.5354	198.2
S33-50-2	343.4	-34.1	5.9	36.95483	197.5
S33-45-3	16.3	10.9	4.4	59.51283	187.7
S33-44-2	4.5	20.2	2.0	68.12455	182.4
S33-44-1	0.2	10.0	4.8	63.13772	182.2
S33-43-2	59.0	50.7	14.0	40.43738	176.4
S33-43-1	359.5	4.0	5.3	60.09873	175.7
S33-41-7	347.7	43.5	3.3	77.40425	174.1
S33-41-5	18.3	31.4	6.3	67.69629	172.6
S33-41-4	328.1	30.4	9.7	57.17792	172.1
S33-41-3	296.0	18.8	6.8	27.10097	171.7

Sample	Ds	Is	MAD	VGP	Deep
S33-41-1	4.6	11.1	4.1	63.35275	169.6
S33-40-3	217.1	-9.1	26.0	-45.8198	165.1
S33-40-2	269.0	60.5	11.4	19.80586	164.3
S33-40-1	14.5	10.8	8.1	60.27224	164.0
S33-39-2	261.3	-9.0	8.6	-9.77229	162.7
S33-38-7	8.4	27.6	18.4	71.12087	156.0
S33-38-5	165.3	-61.0	15.2	-74.5174	153.8
S33-38-4	28.9	28.1	10.5	58.69347	153.3
S33-38-2	89.4	84.0	1.4	31.26286	151.9
S33-37-6	259.7	-28.3	4.5	-16.4964	147.8
S33-37-5	267.8	35.7	1.5	8.511477	145.9
S33-37-4	10.5	30.3	14.4	71.70566	144.9
S33-37-3	122.9	-59.0	13.6	-43.8265	144.3
S33-37-2	240.6	-50.6	8.1	-39.1063	143.9
S33-35-20	8.2	23.8	2.7	69.12757	139.1
S33-35-19	9.9	22.7	7.9	67.94143	137.6
S33-35-18	359.2	10.8	10.0	63.5378	136.0
S33-35-17	5.6	46.9	2.7	83.85084	135.8
S33-35-16	1.9	26.7	3.5	72.13097	135.3
S33-35-15	323.7	15.9	8.3	48.7541	135.0
S33-35-14	18.2	23.4	11.4	64.1595	134.2
S33-35-13	38.7	27.9	5.9	50.87186	133.5
S33-35-12	5.3	23.6	8.6	69.82481	133.2
S33-35-11	6.3	50.3	5.8	84.56252	133.1
S33-35-10	352.1	56.9	4.3	81.43762	132.6
S33-35-9	343.9	15.1	4.6	61.52232	131.2
S33-35-8	356.8	61.9	10.5	78.49959	128.2
S33-35-6	344.8	24.8	8.4	66.51984	126.3
S33-35-5	357.4	33.2	7.9	76.01925	125.7
S33-35-4	360.0	12.3	6.6	64.32168	124.7
S33-35-2	354.7	64.1	5.3	75.47214	122.7
S33-35-1	39.5	47.1	3.2	55.81935	122.4
S33-34-3	350.0	75.9	4.7	57.9428	121.6
S33-34-2	338.0	-0.4	9.7	51.74875	121.3
S33-34-1	66.3	30.3	14.1	28.40811	120.4
S33-33-5	4.4	-4.7	12.9	55.49238	118.0
S33-33-3	324.0	10.5	13.3	47.11015	116.1
S33-33-2	87.0	-56.2	3.9	-16.2973	115.6
S33-33-1	356.4	55.2	6.7	85.14023	114.1
S33-32-1	337.6	9.3	5.0	55.63097	113.0
S33-31-1	356.7	57.1	5.9	83.59963	110.7
S33-30-2	59.0	16.9	5.7	30.77437	104.8
S33-29-2	349.9	4.0	7.0	58.62408	102.9

Sample	Ds	Is	MAD	VGP	Deep
S33-29-1	313.0	3.0	9.1	36.34465	102.2
S33-28-6	324.7	26.1	5.7	53.00666	100.2
S33-28-3	111.0	-3.4	4.5	-18.6509	97.7
S33-28-2	342.8	14.5	7.0	60.70071	95.3
S33-28-1	332.7	55.0	5.4	67.07847	95.0
S33-27-5	291.7	20.0	17.2	23.79379	94.0
S33-27-4	320.0	14.9	2.9	45.5907	93.7
S33-27-3	277.3	15.5	4.4	10.33667	93.0
S33-27-2	305.6	22.2	8.3	36.1483	92.0
S33-27-1	43.0	61.2	5.5	54.57628	91.2
S33-26-6	298.6	20.4	14.3	29.74583	91.0
S33-26-5	45.4	-20.8	18.6	29.1458	90.5
S33-26-4	359.6	43.9	6.7	83.78516	90.2
S33-26-3	6.8	19.4	10.3	67.2002	89.6
S33-26-2	44.1	2.6	11.5	38.42626	89.2
S33-26-1	29.9	13.0	2.6	52.34591	88.4
S33-25-8	44.6	15.4	9.9	42.13926	87.8
S33-25-7	78.6	51.8	5.6	25.1525	87.5
S33-25-6	16.6	27.5	8.8	66.99399	87.2
S33-25-5	334.7	59.9	8.2	67.87335	86.7
S33-25-4	322.4	8.4	6.2	45.21182	86.0
S33-25-2	18.6	16.1	6.7	60.67538	82.9
S33-25-1	10.8	25.1	13.1	68.82291	82.6
S33-24-4	6.4	13.9	8.4	64.44718	82.4
S33-24-3	28.6	4.4	13.6	49.92009	82.3
S33-24-2	345.7	3.9	8.7	57.16036	82.2
S33-23-4	306.5	29.7	5.5	39.09097	81.6
S33-23-3	275.1	48.0	5.1	18.81557	81.2
S33-23-2	17.4	68.6	6.3	66.29992	80.9
S33-23-1	82.8	-76.7	8.2	-25.6109	80.6
S33-22-2	81.4	54.0	14.0	23.83845	75.4
S33-22-1	300.6	4.0	11.5	26.76695	75.2
S33-21-4	323.8	21.0	13.6	50.57101	73.3
S33-21-3	34.6	51.7	7.4	60.82166	73.1
S33-21-2	350.8	54.3	7.4	81.78041	72.8
S33-20-1	348.8	13.4	5.3	62.8056	67.9
S33-19-1	10.2	19.8	9.1	66.34199	66.8
S33-18-1	341.6	19.8	10.4	62.42999	65.4
S33-17-1	45.7	18.1	8.6	42.09806	64.6
S33-16-3	300.1	33.7	8.8	34.8247	63.8
S33-16-2	5.2	27.7	12.6	72.16515	63.7
S33-16-1	315.7	24.3	8.7	45.1583	63.6
S33-15-5	28.8	46.9	11.7	64.85023	62.9

Sample	Ds	Is	MAD	VGP	Deep
S33-15-4	6.9	33.0	8.4	74.7574	62.6
S33-15-3	29.8	65.0	11.6	62.73769	62.2
S33-15-2	48.2	20.8	14.4	40.88427	62.1
S33-15-1	52.4	13.3	13.3	35.2007	61.9
S33-14-4	316.7	46.3	13.4	52.41352	61.5
S33-14-3	25.2	67.3	13.7	63.92447	61.4
S33-14-2	355.2	47.8	4.6	84.87216	61.3
S33-12-9	266.8	-46.4	6.2	-16.7152	59.5
S33-12-7	85.2	14.2	7.8	7.863137	58.5
S33-12-5	35.0	43.2	9.4	58.65977	58.3
S33-12-4	1.7	19.8	5.0	68.24766	58.1
S33-12-3	39.4	58.3	10.4	57.3931	57.9
S33-12-2	340.9	15.6	11.9	60.1801	57.5
S33-11-21	337.6	-30.9	4.6	36.90375	56.8
S33-11-20	309.6	35.3	5.9	43.35239	56.4
S33-11-19	11.4	-24.2	11.8	44.11647	54.9
S33-11-17	6.2	27.5	6.1	71.78686	54.6
S33-11-16	2.6	27.8	6.0	72.70471	54.4
S33-11-5	12.4	-24.9	3.6	43.48996	54.1
S33-11-3	326.1	45.0	14.9	60.06466	53.8
S33-11-2	342.1	18.9	7.5	62.30799	53.6
S33-11-1	345.8	-21.1	8.1	45.07411	53.4
S33-9-8	99.6	61.0	5.8	14.40734	52.5
S33-9-7	171.2	-49.1	13.7	-82.2185	52.1
S33-9-5	338.3	-21.5	6.8	42.20785	51.8
S33-9-4	293.9	-52.6	5.0	-0.07819	51.8
S33-9-3	216.6	-39.6	9.9	-56.2841	51.2
S33-9-2	255.6	7.4	24.6	-10.1626	50.9
S33-8-21	340.4	52.6	6.8	73.45162	50.0
S33-8-19	281.7	56.5	6.8	27.12244	46.0
S33-8-18	346.9	40.4	11.4	75.41351	45.6
S33-8-17	292.1	14.6	8.5	22.64227	45.4
S33-8-16	285.1	23.1	14.6	-17.5606	45.2
S33-8-13	345.4	-6.9	5.6	52.01224	43.0
S33-8-12	356.6	-12.3	7.2	51.74066	42.7
S33-8-11	10.5	-18.5	11.8	47.40183	42.1
S33-8-10	22.0	-32.4	19.5	36.18977	41.2
S33-8-9	24.8	3.3	8.2	51.77489	41.0
S33-8-7	48.1	-27.2	12.0	24.684	40.5
S33-8-6	354.0	-62.3	5.8	14.2986	40.2
S33-8-5	15.8	-1.3	9.5	54.17883	40.1
S33-8-3	80.4	19.4	8.2	13.36028	39.9
S33-8-1	299.8	-81.4	5.3	-22.5602	39.7

Sample	Ds	Is	MAD	VGP	Deep
S33-7-12	347.4	-18.7	11.6	46.78002	39.4
S33-7-11	85.6	23.5	11.3	10.13155	38.6
S33-7-10	258.4	-60.1	10.7	-28.3959	38.4
S33-7-9	259.7	-15.1	14.3	-12.7729	37.8
S33-7-8	106.1	64.0	8.3	12.34974	37.3
S33-7-7	194.6	-3.8	8.8	-56.9973	36.9
S33-7-5	232.9	4.8	16.9	-29.3069	36.6
S33-7-3	208.6	-44.9	8.9	-64.5313	36.2
S33-7-2	97.9	-1.4	8.6	-7.07305	35.9
S33-7-1	338.7	-7.4	3.9	49.03175	29.4
S33-5-11	249.2	-50.9	11.8	-32.2544	26.5
S33-5-9	32.9	50.2	12.7	62.02097	26.1
S33-5-8	18.6	20.0	12.9	62.40456	25.8
S33-5-7	352.9	13.7	8.0	64.18557	25.4
S33-5-6	52.0	47.2	9.5	45.31527	25.1
S33-5-4	350.6	51.3	11.1	82.02485	24.7
S33-5-2	62.1	11.0	12.2	26.52026	24.5
S33-5-1	327.7	65.6	8.4	60.87342	23.8
S33-3-4	356.5	-0.2	10.4	57.8292	16.8
S33-3-2	331.7	19.6	7.3	55.95057	10.3
S33-2-4	351.6	45.7	8.6	81.27579	8.7
S33-2-3	291.5	34.9	11.7	27.89343	8.5
S33-2-2	320.9	19.8	12.4	47.90544	8.2
S33-2-1	329.1	7.5	13.4	49.60631	7.9
S33-1-2	180.5	-13.3	5.0	-64.8365	5.5
S33-1-1	19.6	43.8	13.8	71.73864	3.9

Supplementary Table S2 Spirite isotope data

Sample ID	$\delta^{13}C_c$	$\delta^{18}O_c$	position	Sample ID	$\delta^{13}C_c$	$\delta^{18}O_c$	position
3-1-2	0.4	-20.8	11.3	37-4-2	0.5	-12.6	145.5
3-1-3	-0.6	-16.6	11.3	37-4-1	0.9	-14.2	145.5
31-3	-3.22	-9.59	11.3	37-4	-1.70	-10.98	145.5
3-1-1	-1.5	-15.6	11.3	37-5	-2.26	-9.34	148.9
3-1	-0.10	-14.99	11.3	39-1-2	-2.2	-14.0	160.9
3-2	-1.03	-17.73	11.8	39-1-1	-4.2	-11.6	160.9
4-1	-1.10	-17.71	17.6	39-1	-5.68	-7.09	160.9
4-2	0.01	-17.80	18.0	40-4	-3.42	-5.56	166.1
4-3	0.39	-18.41	18.9	41-1	-3.55	-6.58	169.6
4-3-2	0.5	-18.9	18.9	41-2	-3.29	-7.27	171.1
4-3-1	0.1	-14.9	18.9	43-2-2	-2.2	-7.6	181.7
5-1	-0.29	-16.16	24.2	43-2-1	-1.3	-14.2	181.7
5-2	0.60	-16.37	26.9	44-2	-3.71	-6.68	182.7
6-1	-1.58	-17.23	28.0	45-1	-2.77	-11.71	184.4
7-1	1.68	-16.75	30.8	45-3	-3.80	-8.09	187.6
8-1	-0.97	-17.49	40.9	47-1	-4.49	-7.53	188.6
8-2	-0.44	-17.84	42.7	48-2-1	-2.4	-13.8	191.8
8-3	0.60	-15.73	43.4	48-1	-4.65	-5.39	191.8
8-4	0.29	-15.37	49.6	50-1	-3.94	-6.28	196.1
9-1	-0.25	-16.66	52.0	52-3	-2.43	-14.23	202.1
9-2	1.06	-13.82	52.2	56-2	-3.44	-6.22	211.9
11-1	-0.18	-16.98	53.5	57-1	-2.18	-8.59	217.5
11-2	1.06	-16.57	53.9	59-1	-4.41	-13.29	219.4
11-3	1.66	-17.55	55.6	61-2	-2.70	-8.08	228.5
11-4	1.40	-16.28	57.1	61-5	-4.25	-7.41	230.8
15-1	-1.17	-16.46	62.0	64-1	-2.88	-8.73	237.1
17-1	-2.32	-15.21	64.3	64-2	-3.30	-6.27	237.6
21-1	-3.51	-14.33	72.0	64-4	-3.43	-6.58	238.4
22-1	-3.71	-14.79	75.0	64-5	-3.13	-6.28	239.5
23-1	-3.81	-14.08	79.0	65-1-2	-1.5	-12.8	241.1
25-2-2	-2.5	-12.2	82.9	65-2	-3.23	-5.43	242.2
25-1-1	-2.1	-13.5	82.9	65-3	-1.64	-7.86	244.3
25-1	-0.29	-15.95	82.9	67-1-3	-0.7	-13.4	252.7
27-1	-2.29	-9.73	92.0	67-1-2	-1.2	-13.5	252.7
27-3	-2.41	-9.41	94.3	67-1-1	-1.3	-11.9	252.7
28-2-3	-2.3	-10.0	96.0	69-3	-3.22	-9.85	258.9
28-2-2	-2.8	-11.9	96.0	73-1	-3.12	-8.34	265.9
28-2-1	-2.5	-12.0	96.0	74-1	-4.34	-10.43	270.1
28-2	-3.27	-8.78	96.0	74-2-1	-1.0	-11.4	272.5
28-3	-2.20	-11.36	100.2	75-1	-4.21	-6.25	273.8
28-4	-2.27	-11.54	100.6	75-3	-3.91	-6.20	277.8

Sample ID	$\delta^{13}C_c$	$\delta^{18}O_c$	position	Sample ID	$\delta^{13}C_c$	$\delta^{18}O_c$	position
29-1-3	0.3	-14.9	102.5	76-1	-2.8	-7.5	279.2
29-1-2	0.5	-14.2	102.5	76-1	-3.12	-5.30	279.2
29-1-1	0.7	-13.9	102.5	76-2	-0.47	-6.83	281.1
29-1	-2.26	-15.15	102.5	76-4-2	-2.5	-7.6	282.3
30-1	-2.32	-10.36	106.5	76-4-1	-2.1	-12.8	282.3
31-1	-3.36	-9.41	110.1	76-4	-0.79	-7.96	282.3
32-1	-3.63	-7.99	112.9	76-5	-2.55	-8.29	283.0
33-1	-5.24	-7.87	114.1	78-1-2	-0.6	-7.1	288.2
33-2	-4.34	-6.92	116.1	78-1-1	-6.2	-10.4	288.2
33-3	-3.83	-7.80	116.7	79-1	-3.88	-10.57	289.8
33-4	-2.96	-7.87	117.7	80-1	-3.72	-8.68	291.9
34-1	-2.53	-9.71	121.5	81-2	-3.18	-7.07	297.0
35-2	-4.48	-7.65	122.8	81-3	-3.99	-6.33	297.7
35-4	-3.49	-9.84	125.1	82-2-1	-2.2	-10.7	305.9
35-6	-4.39	-5.54	130.6	82-3	-2.24	-5.40	307.6
35-8-1	-4.5	-12.7	132.9	84-1-2	-3.5	-7.0	314.6
35-9	-3.25	-8.47	135.1	84-1-1	-3.7	-6.2	314.6
35-10	-2.91	-9.18	135.4	84-2	-2.83	-6.61	320.0
36-1	-2.40	-8.55	136.6	86-1	-2.83	-8.17	326.3
37-2	-2.14	-5.58	143.0	86-2	-5.34	-6.03	326.7
37-3	-2.81	-8.81	144.5	86-3	-5.2	-6.7	327.3

Supplementary Table S3 Micrite isotope data

Sample ID	$\delta^{13}C_c$	$\delta^{18}O_c$	$\delta^{18}O_{PDB}$ （20）	$\delta^{18}O_{PDB}$ （30）	$\delta^{18}O_{sw}$ （20）	$\delta^{18}O_{sw}$ （30）	position
86-3	-5.2	-5.4	-33.9	-31.9	-4.0	-2.0	327.3
85-1	-3.6	-6.9	-35.3	-33.4	-5.5	-3.5	320.9
84-1	-3.3	-5.9	-34.4	-32.4	-4.5	-2.5	314.6
83-2	-2.2	-4.8	-33.3	-31.4	-3.4	-1.4	313.9
83-1	-2.0	-3.8	-32.4	-30.4	-2.4	-0.4	305.9
82-2-2	-2.9	-3.9	-32.5	-30.5	-2.6	-0.6	305.9
82-2	-3.3	-3.7	-32.3	-30.3	-2.4	-0.4	305.9
82-1	-4.1	-4.8	-33.4	-31.4	-3.5	-1.5	305.2
81-5	-4.1	-5.0	-33.6	-31.6	-3.7	-1.7	303.9
81-4	-4.6	-3.9	-32.5	-30.5	-2.6	-0.6	303.2
78-1	-4.2	-4.5	-33.1	-31.1	-3.2	-1.2	288.2
77-2	-1.5	-3.5	-32.0	-30.1	-2.1	-0.1	287.3
77-1	-2.6	-3.8	-32.3	-30.4	-2.4	-0.4	286.1
76-3	-2.7	-4.1	-32.6	-30.7	-2.7	-0.7	281.8
75-2	-4.4	-5.7	-34.2	-32.2	-4.3	-2.3	277.2
74-2-2	-1.1	-5.5	-34.0	-32.0	-4.1	-2.1	272.5
72-1	-3.1	-4.7	-33.3	-31.3	-3.4	-1.4	263.5
71-1	-2.4	-5.9	-34.4	-32.4	-4.5	-2.5	262.2
70-1-1	-4.1	-4.7	-33.2	-31.3	-3.3	-1.3	261.0
70-1	-3.5	-5.0	-33.5	-31.6	-3.7	-1.7	261.00
69-1	-3.9	-5.6	-34.1	-32.2	-4.3	-2.3	256.4
68-1	-5.0	-6.4	-34.9	-32.9	-5.0	-3.0	253.3
67-1-4	-4.2	-4.3	-32.9	-30.9	-3.0	-1.0	252.7
67-1	-3.9	-4.4	-32.9	-30.9	-3.0	-1.0	252.7
66-1	-4.1	-5.5	-34.0	-32.1	-4.1	-2.1	245.5
65-1-3	-2.4	-2.1	-30.7	-28.8	-0.8	1.3	241.1
65-1-1	-2.6	-4.1	-32.6	-30.7	-2.7	-0.7	241.1
65-1	-2.6	-3.7	-32.2	-30.3	-2.3	-0.3	241.1
64-3	-3.6	-5.5	-34.0	-32.1	-4.1	-2.1	237.8
63-1	-4.0	-6.4	-34.8	-32.9	-5.0	-3.0	235.4
62-1	-4.3	-6.4	-34.9	-33.0	-5.1	-3.1	232.9
60-2	-3.8	-6.5	-35.0	-33.0	-5.2	-3.2	226.1
59-3	-4.3	-6.2	-34.7	-32.8	-4.9	-2.9	221.6
57-2	-3.9	-6.4	-34.9	-32.9	-5.0	-3.0	217.5
56-4	-3.4	-6.2	-34.7	-32.7	-4.8	-2.8	215.4
55-1	-3.9	-6.0	-34.5	-32.5	-4.6	-2.6	208.8
54-1	-2.5	-5.5	-34.0	-32.1	-4.2	-2.2	208.1
53-1	-4.2	-6.6	-35.1	-33.2	-5.3	-3.3	206.1
52-1	-3.6	-6.0	-34.5	-32.5	-4.6	-2.6	200.2
51-1	-3.7	-6.1	-34.5	-32.6	-4.7	-2.7	197.8
50-1	-3.9	-6.2	-34.7	-32.8	-4.9	-2.9	196.1

Sample ID	$\delta^{13}C_c$	$\delta^{18}O_c$	$\delta^{18}O_{PDB}$（20）	$\delta^{18}O_{PDB}$（30）	$\delta^{18}O_{sw}$（20）	$\delta^{18}O_{sw}$（30）	position
49-2	-2.8	-5.4	-33.9	-32.0	-4.1	-2.1	194.5
48-2-2	-4.9	-4.5	-33.1	-31.1	-3.2	-1.2	191.8
45-1	-4.6	-6.7	-35.2	-33.2	-5.3	-3.3	184.4
44-1	-3.8	-4.0	-32.5	-30.6	-2.6	-0.6	182.2
43-1	-3.8	-5.4	-33.9	-32.0	-4.0	-2.0	181.7
42-2	-3.4	-4.7	-33.2	-31.3	-3.3	-1.3	175.3
42-1	-5.0	-3.8	-32.4	-30.4	-2.4	-0.4	174.5
41-3	-4.1	-5.9	-34.4	-32.4	-4.5	-2.5	174.0
40-2	-3.8	-5.5	-34.0	-32.1	-4.2	-2.2	164.2
39-1-3	-5.7	-6.9	-35.3	-33.4	-5.5	-3.5	160.9
38-4	-3.5	-3.8	-32.3	-30.4	-2.4	-0.4	157.4
38-1	-5.3	-4.8	-33.3	-31.4	-3.5	-1.4	151.4
37-1	-4.1	-4.5	-33.0	-31.1	-3.1	-1.1	141.5
37-1	-2.5	-4.5	-33.0	-31.0	-3.1	-1.1	141.5
36-2	-3.2	-6.2	-34.7	-32.7	-4.9	-2.9	138.4
35-8-2	-4.9	-4.0	-32.5	-30.6	-2.6	-0.6	132.9
35-8	-5.0	-5.0	-33.5	-31.5	-3.6	-1.6	132.9
average	-3.7	-5.1	-33.6	-31.7	-3.8	-1.8	

Supplementary Figure S1

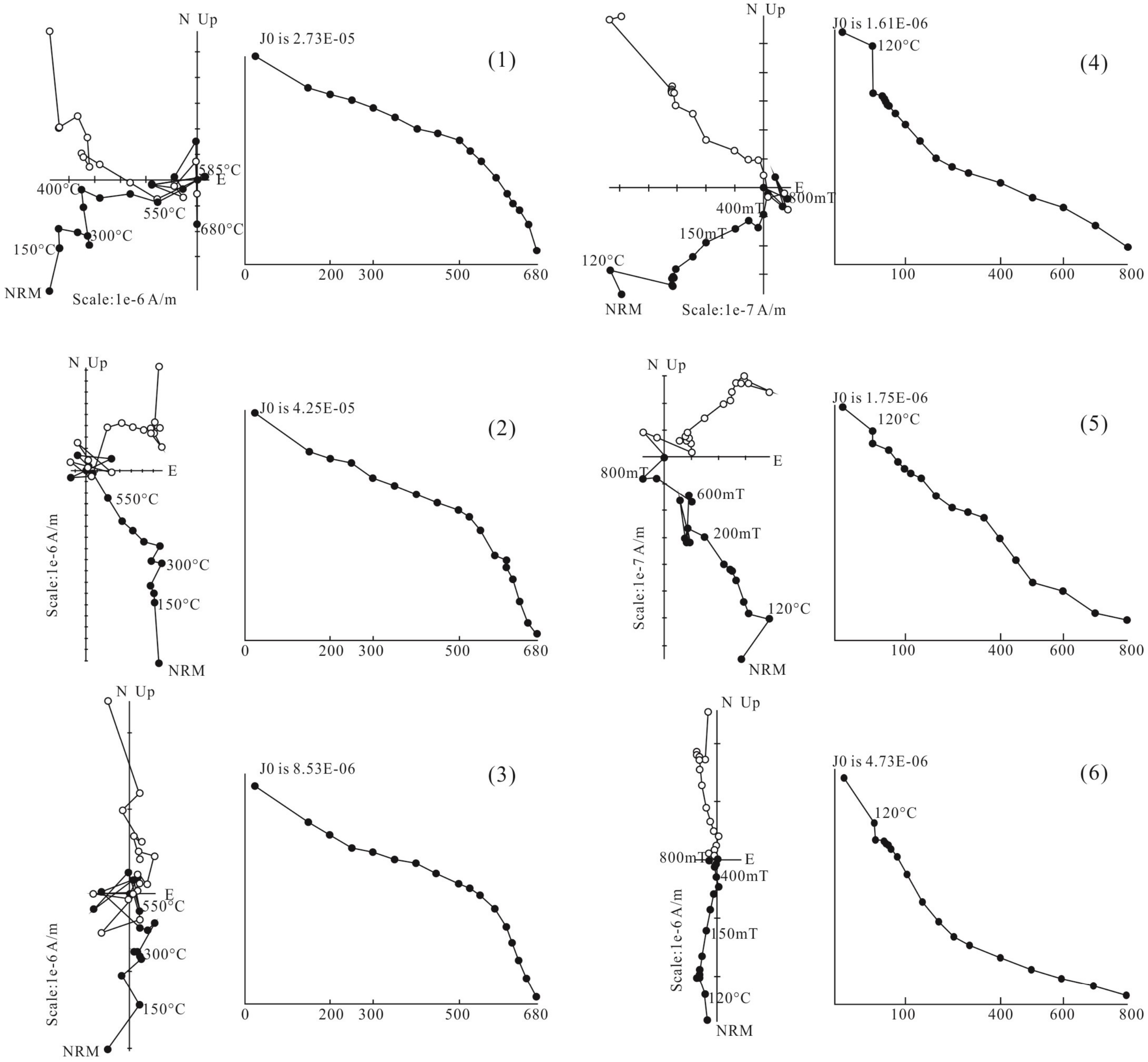

○ Declination

● Inclination

Stepwise thermal demagnetization (1-3) and alternating demagnetization (4-6)diagrams in-situ of representative samples from the Gerze section.

Figure S1 Typical demagnetization curves
